# Deciphering the Functioning of Microbial Communities: Shedding Light on the Critical Steps in Metaproteomics

**DOI:** 10.3389/fmicb.2019.02395

**Published:** 2019-10-24

**Authors:** Augustin Géron, Johannes Werner, Ruddy Wattiez, Philippe Lebaron, Sabine Matallana-Surget

**Affiliations:** ^1^Division of Biological and Environmental Sciences, Faculty of Natural Sciences, University of Stirling, Stirling, United Kingdom; ^2^Department of Proteomic and Microbiology, University of Mons, Mons, Belgium; ^3^Department of Biological Oceanography, Leibniz Institute for Baltic Sea Research, Rostock, Germany; ^4^Sorbonne Universités, UPMC Université Paris 06, USR 3579, LBBM, Observatoire Océanologique, Banyuls-sur-Mer, France

**Keywords:** metaproteomics, metagenomics, bioinformatics, mass spectrometry, microbial ecology

## Abstract

Unraveling the complex structure and functioning of microbial communities is essential to accurately predict the impact of perturbations and/or environmental changes. From all molecular tools available today to resolve the dynamics of microbial communities, metaproteomics stands out, allowing the establishment of phenotype–genotype linkages. Despite its rapid development, this technology has faced many technical challenges that still hamper its potential power. How to maximize the number of protein identification, improve quality of protein annotation, and provide reliable ecological interpretation are questions of immediate urgency. In our study, we used a robust metaproteomic workflow combining two protein fractionation approaches (gel-based versus gel-free) and four protein search databases derived from the same metagenome to analyze the same seawater sample. The resulting eight metaproteomes provided different outcomes in terms of (i) total protein numbers, (ii) taxonomic structures, and (iii) protein functions. The characterization and/or representativeness of numerous proteins from ecologically relevant taxa such as *Pelagibacterales*, *Rhodobacterales*, and *Synechococcales*, as well as crucial environmental processes, such as nutrient uptake, nitrogen assimilation, light harvesting, and oxidative stress response, were found to be particularly affected by the methodology. Our results provide clear evidences that the use of different protein search databases significantly alters the biological conclusions in both gel-free and gel-based approaches. Our findings emphasize the importance of diversifying the experimental workflow for a comprehensive metaproteomic study.

## Introduction

Metaproteomics aims at characterizing the total proteins obtained from microbial communities (Wilmes and Bond, 2004) and, in association with metagenomics, unraveling the functional complexity of a given ecosystem (Franzosa et al., 2015). Since the first environmental metaproteomic study performed in the Chesapeake Bay (Kan et al., 2005), numerous investigations were carried out in a variety of environments using descriptive, comparative, and/or quantitative approaches (Matallana-Surget et al., 2018). Comparative metaproteomics was often used to describe spatial and seasonal changes in aquatic ecosystems using (i) *in situ* (Morris et al., 2010; Teeling et al., 2012; Williams et al., 2012; Georges et al., 2014), (ii) mesocosms (Lacerda and Reardon, 2009; Bryson et al., 2016), or (iii) microcosms (Russo et al., 2016) approaches.

Metaproteomics on marine ecosystems is a rapidly expanding field that involves a series of challenging steps and critical decisions in its workflow (Wilmes et al., 2015; Heyer et al., 2017; Matallana-Surget et al., 2018; Saito et al., 2019). The marine metaproteomic workflow consists mainly of four steps: (i) sampling and protein extraction, (ii) protein separation, (iii) mass spectrometry, and (iv) protein identification/annotation (Wöhlbrand et al., 2013). Until now, standardized experimental protocols are still missing, leading to methodological inconsistencies and data interpretation biases across metaproteomic studies (Leary et al., 2013; Tanca et al., 2013; Timmins-Schiffman et al., 2017).

Protein identification strongly relies on both the quality of experimental mass spectra (MS) and the comprehensiveness of the protein search database (DB) (Wöhlbrand et al., 2013). Both gel-based (Wang et al., 2014) and shotgun gel-free (Morris et al., 2010; Saito et al., 2015) approaches have been used in metaproteomic analyses and both were found to be complementary (Matallana-Surget et al., 2018). Two main data sources are commonly used to construct protein search DB: public protein repositories, and/or metagenomic data (Heyer et al., 2017). Identifying proteins by searching against public protein repositories such as UniProtKB/SwissProt, UniProtKB/TrEMBL, UniRef, NCBI, or Ensembl is challenging because of the large size of these DBs, which increase search space and overestimate false discovery rate (FDR), thus decreasing the total number of identified proteins (Nesvizhskii, 2010; Jagtap et al., 2013; Tanca et al., 2013; Timmins-Schiffman et al., 2017). To address the issue of large size DB, different strategies were developed such as pseudo-metagenome approach (Heyer et al., 2017), partial searches against smaller sub-DB (Muth et al., 2015a; Tanca et al., 2016), or the two-round DB searching method (Jagtap et al., 2013). The two-round DB searching method consists in searching experimental MS against a refined database composed of the protein sequences identified in a preliminary error tolerant search, allowing significant increase in the total number of identified proteins. This strategy was extensively used in recent metaproteomics studies (Russo et al., 2016; Serrano-Villar et al., 2016; Deusch et al., 2017; Gallois et al., 2018). Regarding metagenomic data, both assembled (Teeling et al., 2012) and non-assembled (Herbst et al., 2016; Tanca et al., 2016) sequencing reads were used in metaproteomics for protein search DB creation. Skipping read assembly was shown to prevent information loss and potential noise introduction and led to higher protein identification yield (May et al., 2016).

Metaproteomic data analysis also involves taxonomic and functional annotation. Due to the protein inference issue (i.e., a same peptide can be found in homologous proteins), inaccurate protein annotations are commonly encountered in metaproteomics (Herbst et al., 2016). To overcome this issue, protein identification tools such as Pro Group algorithm (Absciex, 2014), Prophane (Schneider et al., 2011), or MetaProteomeAnalyzer (Muth et al., 2015b) automatically group homologous protein sequences. In our study, we used the mPies tool (Werner et al., 2019), which uses sequence-based alignment to compute taxonomic consensus annotation on protein groups using last common ancestor (LCA) (Huson et al., 2016; Heyer et al., 2017). mPies also provides a novel consensus functional annotation using UniProt, that gives more accurate insights into the diversity of protein functions compared to former strategies mapping proteins on broader functional categories, such as KEGG (Kanehisa et al., 2018) or COGs (Galperin et al., 2015).

To what extend the methodology affects the metaproteome interpretation has already been studied in artificial microbial communities (Tanca et al., 2013) and gut microbiomes (Tanca et al., 2016; Rechenberger et al., 2019) but its impact on marine samples still remains poorly documented (Timmins-Schiffman et al., 2017). In this study, we used a robust experimental design comparing the combined effect of protein search DB choice and protein fractionation approach on the same sea surface sample. For this purpose, two sets of peptide spectra resulting from gel-based and gel-free approaches were searched against four DBs derived from the same raw metagenomic data. The resulting eight metaproteomes were quantitatively and qualitatively compared, demonstrating to which extent diversifying metaproteomic workflow allows the most comprehensive understanding of microbial communities dynamics.

## Materials and Methods

### Sampling

Seawater samples (*n* = 4) were collected in summer (June 2014) at the SOLA station, located 500 m offshore of Banyuls-sur-Mer, in the Northwestern Mediterranean Sea (42° 49′N, 3° 15′W). Each sample consisted of 60 L of sea surface water, pre-filtered at 5 μm and subsequently sequentially filtered through 0.8 and 0.2 μm pore-sized filters (polyethersulfone membrane filters, PES, 142 mm, Millipore). Four independent sets of filters were obtained and flash frozen into liquid nitrogen before storage at −80°C.

### Protein Isolation for Gel-Based and Gel-Free Approaches

A combination of different mechanical (sonication/freeze–thaw) and chemical (urea/thiourea containing buffers, acetone precipitation) extraction techniques were used on the filtered seawater samples to maximize the recovery of protein extracts from the filters. The 0.2 μm filters were removed from their storage buffer and cut into quarters using aseptic procedures. Protein isolation was performed on four 0.2 μm filters. The same protein isolation protocol was used for both gel-based and gel-free approaches. The filters were suspended in a lysis buffer containing 8 M urea/2 M thiourea, 10 mM HEPES, and 10 mM dithiothreitol (DTT). Filters were subjected to five freeze–thaw cycles in liquid N_2_ to release cells from the membrane. Cells were mechanically broken by sonication on ice (five cycles of 1 min with tubes on ice, amplitude 40%, 0.5 pulse rate) and subsequently centrifuged at 16,000 × *g* at 4°C for 15 min. To remove particles that did not pellet during the centrifugation step, we filtered the protein suspension through a 0.22 μm syringe filter and transferred into a 3 kDa cutoff Amicon Ultra-15 filter unit (Millipore) for protein concentration. Proteins were precipitated with cold acetone overnight at −80°C, with an acetone/aqueous protein solution ratio of 4:1. Total protein concentration was determined by a Bradford assay, according to the Bio-Rad Protein Assay kit (Bio-Rad, Hertfordshire, United Kingdom) according to the manufacturer’s instructions, with bovine γ-globulin as a protein standard. Protein samples were reduced with 25 mM DTT at 56°C for 30 min and alkylated with 50 mM iodoacetamide at room temperature for 30 min. For gel-free liquid chromatography tandem mass spectrometry analysis, a tryptic digestion (sequencing grade modified trypsin, Promega) was performed overnight at 37°C, with an enzyme/substrate ratio of 1:25.

### Gel-Based Proteomics Approach

Protein isolates diluted in Laemmli buffer (2% SDS, 10% glycerol, 5% β-mercaptoethanol, 0.002% bromophenol blue, and 0.125 M Tris–HCl, pH 6.8) and sonicated in a water bath six times for 1 min at room temperature. After 1 min incubation at 90°C, the protein solutions were centrifuged at 13,000 rpm at room temperature for 15 min. The SDS-PAGE of the protein mixtures was conducted using 4–20% precast polyacrylamide mini-gels (Pierce). The protein bands were visualized with staining using the Imperial Protein Stain (Thermo) according to the manufacturer’s instructions. The corresponding gel lane containing proteins was cut in 17 pieces of 1 mm each. Enzymatic digestion was performed by the addition of 10 μl modified sequencing grade trypsin (0.02 mg/ml) in 25 mM NH_4_HCO_3_ to each gel piece. The samples were placed for 15 min at 4°C and incubated overnight at 37°C. The reaction was stopped with 1 μl 5% (v/v) formic acid. Tryptic peptides were analyzed by liquid chromatography tandem mass spectrometry.

### Liquid Chromatography Tandem Mass Spectrometry Analysis

Purified peptides from digested protein samples from gel-free and gel-based proteomics were identified using a label-free strategy on an UHPLC-HRMS platform composed of an Eksigent 2D liquid chromatograph and an AB SCIEX Triple TOF 5600. Peptides were separated on a 25 cm C18 column (Acclaim pepmap 100, 3 μm, Dionex) by a linear acetonitrile (ACN) gradient [5–35% (v/v), in 15 or 120 min] in water containing 0.1% (v/v) formic acid at a flow rate of 300 nL min^–1^. MS were acquired across 400–1,500 *m*/*z* in high-resolution mode (resolution >35,000) with 500 ms accumulation time. Six microliters of each fraction were loaded onto a pre-column (C18 Trap, 300 μm i.d. × 5 mm, Dionex) using the Ultimate 3000 system delivering a flow rate of 20 μl/min loading solvent [5% (v/v) ACN, 0.025% (v/v) TFA]. After a 10 min desalting step, the pre-column was switched online with the analytical column (75 μm i.d. × 15 cm PepMap C18, Dionex) equilibrated in 96% solvent A [0.1% (v/v) formic acid in HPLC-grade water] and 4% solvent B [80% (v/v) ACN, 0.1% (v/v) formic acid in HPLC-grade water]. Peptides were eluted from the pre-column to the analytical column and then to the mass spectrometer with a gradient from 4 to 57% solvent B for 50 min and 57 to 90% solvent B for 10 min at a flow rate of 0.2 μL min^–1^ delivered by the Ultimate pump. Positive ions were generated by electrospray and the instrument was operated in a data-dependent acquisition mode described as follows: MS scan range: 300–1,500 *m*/*z*, maximum accumulation time: 200 ms, ICC target: 200,000. The top four most intense ions in the MS scan were selected for MS/MS in dynamic exclusion mode: ultrascan, absolute threshold: 75,000, relative threshold: 1%, excluded after spectrum count: 1, exclusion duration: 0.3 min, averaged spectra: 5, and ICC target: 200,000. Gel-based and gel-free metaproteomic data were submitted to iProx (Ma et al., 2018) (Project ID: IPX0001684000/PXD014582).

### Databases Creation and Protein Identification

Protein searches were performed with ProteinPilot (ProteinPilot Software 5.0.1; Revision: 4895; Paragon Algorithm: 5.0.1.0.4874; AB SCIEX, Framingham, MA, United States) (Matrix Science, London, United Kingdom; v. 2.2). Paragon searches 34 were conducted using LC MS/MS Triple TOF 5600 System instrument settings. Other parameters used for the search were as follows: Sample Type: Identification, Cys alkylation: Iodoacetamide, Digestion: Trypsin, ID Focus: Biological Modifications and Amino acid substitutions, Search effort: Thorough ID, Detected Protein Threshold [Unused ProtScore (Conf)]>: 0.05 (10.0%).

Three DBs were created using the same metagenome (EMBL-EBI Project number: ERP009703, Ocean Sampling Day 2014, sample: OSD14_2014_06_2m_NPL022, run ID: ERR771073) (MiSeq Illumina Technology) and were generated with mPies v 0.9, our recently in house developed mPies program freely available at https://github.com/johanneswerner/mPies/ (Supplementary Presentation 1; Werner et al., 2019). The three DBs were: (i) a non-assembled metagenome-derived DB (NAM-DB), (ii) an assembled metagenome-derived DB (AM-DB), and (iii) a taxonomy-derived DB (TAX-DB) (Table 1). Briefly, mPies first trimmed sequencing raw reads with Trimmomatic (Bolger et al., 2014). For NAM-DB, mPies directly predicted genes from trimmed sequencing reads with FragGeneScan (Rho et al., 2010). For AM-DB, mPies first assembled trimmed sequencing reads into contigs using metaSPAdes (Nurk et al., 2017) and subsequently called genes with Prodigal (Hyatt et al., 2010). For TAX-DB, mPies created a pseudo-metagenome using SingleM (Woodcroft, 2018) to predict operational taxonomic units from the trimmed sequencing reads and retrieved all the taxon IDs at genus level. All available proteomes for each taxon ID were subsequently downloaded from UniProtKB/TrEMBL. Duplicated protein sequences were removed with CD-HIT (Fu et al., 2012) from each DB.

**TABLE 1:** Two-round search performances obtained for each methodology.

	**Database**	**Number of proteins**	**Number of peptide**	**Coverage of peptide**	**Number of distinct**	**Number of proteins**
		**in database**	**spectra identified^a^**	**spectra identified (%)**	**peptides Identified^a^**	**after validation^a,b^**
**First-round searches**						
Gel-free	AM-DB	64,613	24,684	7.8	3,237	347
	NAM-DB	462,821	35,430	11.1	4,295	834
	TAX-DB	13,426,277	10,626	3.3	1,624	607
Gel-based	AM-DB	64,613	2,066	24.7	1,408	201
	NAM-DB	462,821	2,584	30.9	1,849	652
	TAX-DB	13,426,277	2,304	27.5	1,526	496
**Second-round searches**						
Gel-free	AM-DB	782	42,831	13.4	8,487	549
	NAM-DB	4,277	57,840	18.2	9,113	1,131
	TAX-DB	18,480	31,700	10.0	4,497	464
	Comb-DB	23,405	56,530	17.7	8,273	1,048
Gel-based	AM-DB	377	2,619	31.3	1,815	277
	NAM-DB	3,080	2,897	34.6	2,034	714
	TAX-DB	19,036	2,951	35.3	1,777	434
	Comb-DB	22,493	3,684	44.0	2,244	700

***^a^**Values comprised in 95% confidence interval. **^b^**Values at 1% global FDR and after manual validation for proteins identified with one peptide. Searching parameters are provided in the section “Materials and Methods.”*

Gel-based and gel-free MS/MS spectra were individually searched twice against the DBs. In the first-round search, full size NAM-DB, AM-DB, and TAX-DB were used (Table 1). In the second-round search, each DB was restricted to the protein sequences identified in the first-round search. For both gel-free and gel-based approaches, the second round NAM-DB, AM-DB, and TAX-DB were merged and redundant protein sequences were removed, leading to two combined DBs (Comb-DBs), subsequently searched against gel-based and gel-free MS/MS spectra. Consequently, a total of eight metaproteomes obtained from four DBs: NAM-DB, AM-DB, TAX-DB, and Comb-DB were analyzed in this paper. A FDR threshold of 1%, calculated at the protein level, was used for each protein searches. Proteins identified with one single peptide were validated by manual inspection of the MS/MS spectra, ensuring that a series of at least five consecutive sequence-specific b-and y-type ions was observed.

### Protein Annotation

Identified proteins were annotated using mPies. For taxonomic and functional annotation, mPies used Diamond (Buchfink et al., 2015) to align each identified protein sequences against the non-redundant NCBI DB and the UniProt DB (Swiss-Prot), respectively, and retrieved up to 20 best hits based on alignment score (>80). For taxonomic annotation, mPies returned the LCA among the best hits via MEGAN (bit score >80) (Huson et al., 2016). For functional annotation, mPies returned the most frequent protein name, with a consensus tolerance threshold >80% of similarity among the 20 best blast hits. Proteins annotated with a score below this threshold were manually validated. Manual validation was straightforward as the main reasons leading to low annotation score were often explained by the characterization of protein isoforms or different sub-units of the same protein. To facilitate the understanding of this annotation step, examples were provided in Supplementary Presentation 2. Annotated proteins files are available in Supplementary Data Sheet 1.

## Results and Discussion

### Database Choice Affects the Total Number of Protein Identification

The two-rounds search strategy commonly used in recent metaproteomics studies (Russo et al., 2016; Serrano-Villar et al., 2016; Deusch et al., 2017; Gallois et al., 2018) significantly reduced the size of protein search DBs, which in turn increased the total number of identified proteins with both AM-DB and NAM-DB (Table 1). Overall, the total number of identified proteins was found to be consistent with other metaproteomics studies conducted in marine oligotrophic waters (Morris et al., 2002; Sowell et al., 2009; Williams et al., 2012, 2013; Dong et al., 2014). NAM-DB led to greater protein identifications (gel-based: 714, gel-free: 1,131) than AM-DB (gel-based: 277 and gel-free: 549) and TAX-DB (gel-based: 434 and gel-free: 464) for both proteomics approaches. Comb-DB gave comparable results than NAM-DB in both approaches (gel-based: 700 and gel-free: 1,048). In AM-DB approach, the assembly process involved the removal of reads that cannot be assembled into longer contigs, leading to loss of gene fragments and consequently fewer identified proteins (Cantarel et al., 2011). As high proportions of prokaryotic genomes are protein-coding, gene fragments can directly be predicted from non-assembled sequencing reads (Koonin, 2009). TAX-DB suffered from a reduction of protein detection sensitivity due to its large size in the first round search, which negatively influenced FDR statistics and protein identification yield (Jagtap et al., 2013).

### Protein Search DB Affects the Taxonomic Structure

**FIGURE 1:**
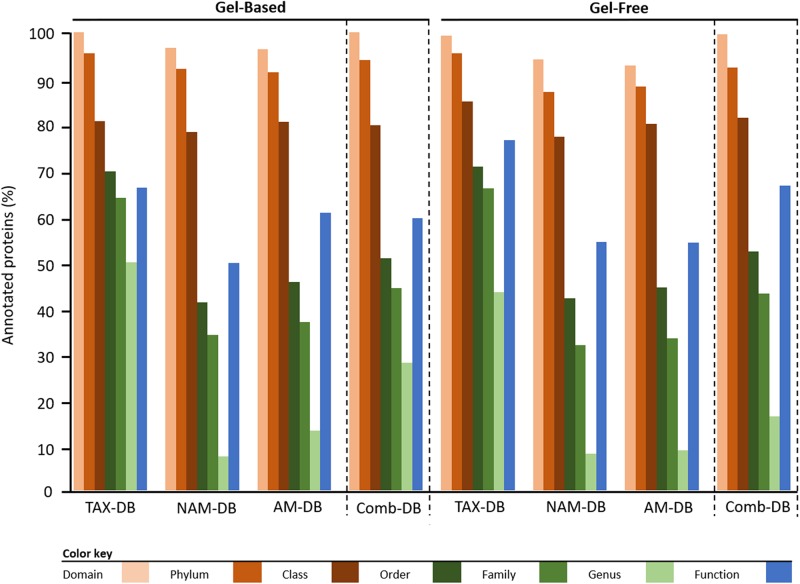
Taxonomic and functional protein annotation. Comparison of the proportion of proteins for which a consensus annotation was found. Bars represent the percentage of annotated proteins versus the total identified proteins depending on methodology.

The proportion of proteins, for which a LCA was found, decreased with lowering taxonomic hierarchy (Domain > Phylum > Class > Order > Family > Genus), independently of the methodology (Figure 1). The proportion of annotated proteins at the domain, phylum and class levels remained constant with an average of 97.3 ± 1.0, 92.0 ± 1.1, and 80.3 ± 0.8%, respectively (Figure 1 and Supplementary Table 1). At order level and below, TAX-DB performed the best at assigning a LCA, in both gel-free and gel-based approaches. These results can be explained by the fact that proteins were annotated using sequence-based alignment method (Werner et al., 2019). TAX-DB comprised complete protein sequences from UniProtKB, which allowed accurate annotations. This result confirmed that LCA approach performed at the protein level is affected by DB, as it was previously demonstrated at the peptide level (May et al., 2016).

At phylum level, most of the proteins identified were assigned to *Proteobacteria* and the least abundant were mainly assigned to *Bacteroidetes* and *Cyanobacteria* (Table 2). Although *Proteobacteria* showed similar proportion in all metaproteomes (90.9 ± 0.97%), the representativeness of *Bacteroidetes* and *Cyanobacteria* was found to be more variable across the different DBs. The similar distribution can be explained by the fact that the three DBs used in this study were derived from the same metagenome. Indeed, by using distinct data sources (metagenomes and different public repositories), contrasting distributions can be anticipated, as it was recently demonstrated (Timmins-Schiffman et al., 2017). In our study, *Alphaproteobacteria* were found to be the most represented class (72.9 ± 1.9%) followed by *Gammaproteobacteria* (18.2 ± 2.0%), *Flavobacteriia* (4.1 ± 0.5%), and unclassified *Cyanobacteria* (3.0 ± 0.7%) (Table 2). The dominance of *Alpha-* and *Gammaproteobacteria* was often reported in other marine metaproteomic studies (Morris et al., 2010; Williams et al., 2012; Georges et al., 2014) due to their high distribution in most marine sampling sites. Other studies focusing on sea surface sample also supported the presence of *Cyanobacteria* (Sowell et al., 2009) and *Flavobacteriia* (Williams et al., 2013).

**TABLE 2:** Comparison of the distribution of proteins assigned at phylum and class levels for each methodology.

	**Gel-based**				**Gel-free**			
	**TAX-DB**	**NAM-DB**	**AM-DB**	**Comb-DB**	**TAX-DB**	**NAM-DB**	**AM-DB**	**Comb-DB**
**Phylum**								
Proteobacteria	87.2	92.7	95.2	89.8	87.6	92.9	91.5	90.0
Cyanobacteria	6.3	2.9	2.0	4.0	2.6	4.8	1.6	2.0
Bacteroidetes	4.9	4.2	2.4	5.0	7.6	1.4	6.1	5.8
Other (<1%)	1.6	0.2	0.4	1.2	2.2	0.9	0.8	2.2
**Class**								
Alphaproteobacteria	73.7	81.6	79.9	74.3	69.7	68.4	68.4	67.5
Gammaproteobacteria	12.9	11.5	14.8	14.4	18.0	25.7	24.4	23.5
Flavobacteriia	6.5	3.7	2.2	3.9	6.3	3.5	4.9	4.8
Unclassified Cyanobacteria	3.9	2.7	1.8	5.3	2.5	1.4	1.2	2.1
Other (<1%)	3.0	0.5	1.2	2.1	3.6	1.0	1.0	2.2

*Values represent the proportion of proteins with identical taxonomy on total identified protein using TAX-DB, NAM-DB, AM-DB, or Comb-DB in both gel-free and gel-based approaches. The number of peptides detected for each protein was used as quantitative value. Taxa displaying a proportion <1% were gathered into “Other” category.*

**FIGURE 2:**
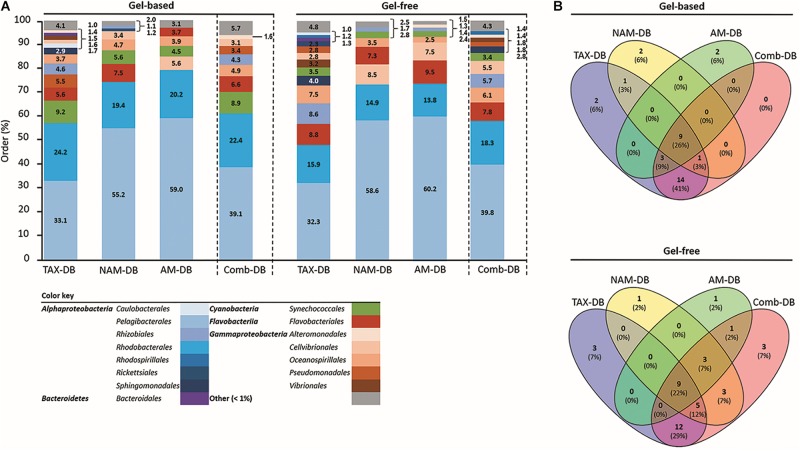
**(A)** Relative taxonomic composition at order level for each methodology. Values represent the proportion of proteins with identical taxonomy on total identified protein using TAX-DB, NAM-DB, AM-DB, or Comb-DB in both gel-free and gel-based approaches. The number of peptides detected for each protein was used as quantitative value. Taxa displaying a proportion <1% were gathered into “Other” category. **(B)** Venn diagrams showing the number of common and unique taxa identified at order level.

At the order level and below, the choice of DB was found to affect both qualitatively and quantitatively the taxonomic distribution, independently of the protein fractionation approach (Figure 2 and Supplementary Figures 1, 2). Although *Pelagibacterales* and *Rhodobacterales* were found to be the most dominant taxa independently of the methodology, *Pelagibacterales* were found to represent >50% of the total annotated proteins in both NAM-DB and AM-DB (Figure 2A). *Pelagibacterales* are comprised of the most dominant marine microorganisms in the oceans (Morris et al., 2002) and the dominance of this order in all metaproteomes was in line with prior sea surface metaproteomic studies (Sowell et al., 2009, 2011; Morris et al., 2010; Williams et al., 2012; Georges et al., 2014). The observation of high protein expression profiles assigned to *Rhodobacterales* was also previously reported (Dong et al., 2014). *Flavobacteriales* were overall more represented in the gel-free approach as well as *Cellvibrionales* but only with NAM-DB and AM-DB. *Synechococcales* were more frequently identified in the metaproteomes obtained from the gel-based approach. TAX-DB led to the characterization of many proteins from the following taxa: *Pseudomonadales*, *Rhizobiales*, and *Sphingomonadales*. These taxa were either absent or rarely represented in NAM-DB or AM-DB. As stated above, TAX-DB provided the highest number of annotated proteins, explaining the more diverse distribution obtained using this DB. Interestingly, the taxonomic distributions obtained with Comb-DB were found to be a good compromise between TAX-DB, NAM-DB, and AM-DB (Figure 2A). As shown in the Venn diagrams provided in Figure 2B, only one quarter out of the 34 and 41 unique orders observed in gel-based and gel-free approaches, respectively, was common to all DBs. Around 40 and 30% of unique orders were exclusively characterized in TAX-DB and Comb-DB in gel-based and gel-free approaches, respectively, demonstrating the performance of those DBs at extracting the broadest diversity.

### Proteomics Workflow and Protein Search DB Affect Functional Identification

The total number of proteins, for which a functional consensus annotation was found, decreased with the following order: TAX-DB (gel-based: 66%, gel-free: 77%) > AM-DB (gel-based: 61%, gel-free: 54%) > NAM-DB (gel-based: 50%, gel-free: 54%) (Figure 1 and Supplementary Table 1). Using Comb-DBs, 59 and 67% of functional annotation were observed in gel-based and gel-free approach, respectively. Alignment-based functional annotation (Werner et al., 2019) might be sub-optimal when protein architecture is different. In that case, domain prediction using InterProScan (Jones et al., 2014) would be a complementary approach that would confirm an alignment-based functional consensus.

**FIGURE 3:**
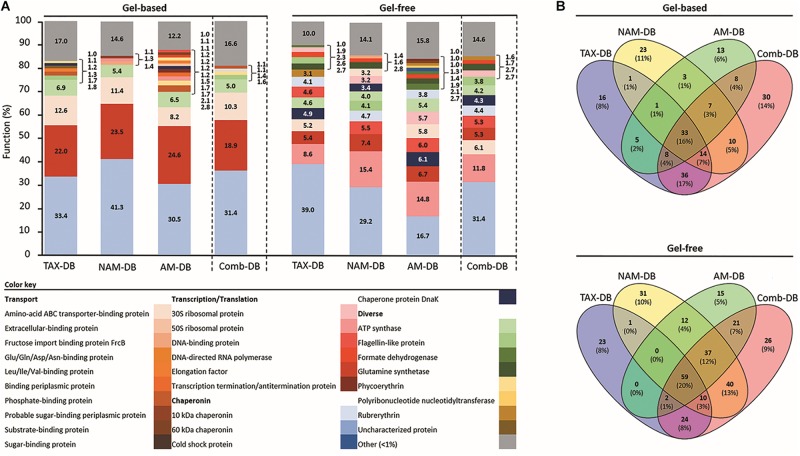
**(A)** Relative functional composition for each methodology. Values represent the proportion of proteins with identical functional name on total identified protein using TAX-DB, NAM-DB, AM-DB, or Comb-DB in both gel-free and gel-based approaches. The number of peptides detected for each protein was used as quantitative value. Protein isoforms and/or sub-units were grouped under the same function. Functions displaying a proportion <1% were gathered into “Other” category. **(B)** Venn diagrams showing the number of common and unique protein functions.

In all metaproteomes, the 60 kDa chaperonin was found to be the most abundant protein (Figure 3A). The prevalence of chaperonin proteins was previously observed in other marine metaproteomic studies (Sowell et al., 2009, 2011; Williams et al., 2012). The 60 kDa chaperonin is an essential protein involved in large range of protein folding and could potentially act as signaling molecule (Maguire et al., 2002). Moreover, this protein is found in nearly all bacteria. Some taxa, such as *Alphaproteobacteria* or *Cyanobacteria*, often contain several 60 kDa chaperonin homologs (Lund, 2009). On top of its ubiquity and its vital role, the abundance of the 60 kDa chaperonin could be interpreted as a response to environmental stresses exposure (Sowell et al., 2009, 2011; Williams et al., 2012).

Protein fractionation (gel-based versus gel-free) was found to affect both qualitatively and quantitatively the functional distribution as shown in Figure 3. The gel-free approach provided the greatest diversity of protein functions in comparison to the gel-based approach (Figure 3A). Only 16 and 20% of the protein functions were found to be common in all DBs from the gel-based and gel-free approaches, respectively (Figure 3B). In the gel-based approach, three main functions namely the elongation factor protein, the amino-acid ABC transporter-binding protein, and the ATP synthase were observed in all DBs (Figure 3A). In contrast, in the gel-free approach, a higher number of abundant proteins was observed, including: 50S ribosomal proteins, elongation factor protein, ATP synthase, DNA-binding protein, amino-acid ABC transporter-binding protein, 10 kDa chaperonin, and the chaperone protein DnaK (Figure 3A). In both proteomics approaches, each individual DB allowed the characterization of a significant number of unique protein functions (Figure 3B). Comb-DB proved to be effective at merging the results obtained from each individual DB, leading to the highest number of identified functions.

### Metaproteomic Workflow Alters Biological Interpretation

**FIGURE 4:**
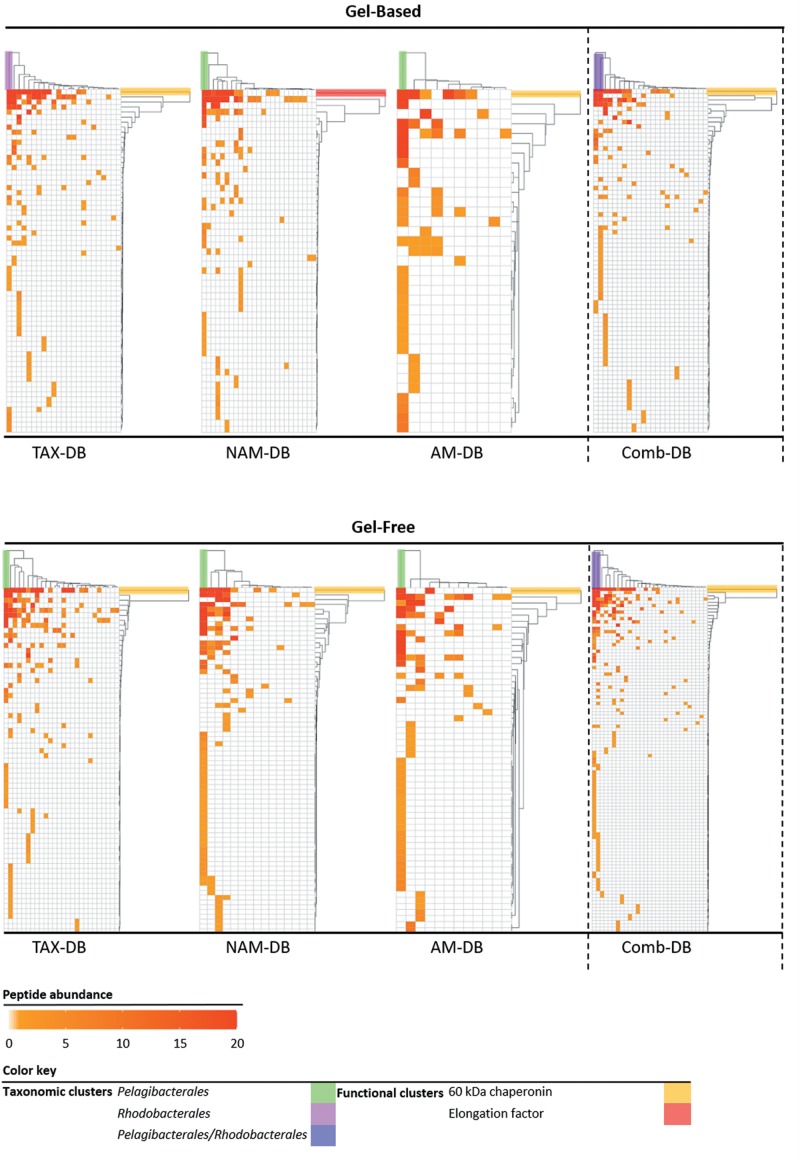
Heatmaps of the taxonomic (top clusters) and the functional (right clusters) linkages for each methodology. Proteins annotated at both order and functional levels were ranked according to the number of identified peptides. Protein isoforms and/or sub-unit were grouped under the same function. Clusters were determined using complete linkage hierarchical clustering and Euclidean distance metric.

All proteins annotated at both taxonomic (order rank) and functional levels were clustered and visualized into heatmaps for each DB (Figure 4). Interestingly, in five out of six heatmaps derived from NAM-DB, AM-DB, and TAX-DB, *Pelagibacterales* was found to be a taxonomic cluster that stood out from all other taxa comprising of *Rhodobacterales*, *Rhizobiales*, *Pseudomonadales*, *Oceanospirillales*, *Cellvibrionales*, *Flavobacteriales*, or *Synechococcales*. An exception was observed for TAX-DB in the gel-based approach where *Rhodobacterales* formed a distinct cluster instead of *Pelagibacterales*. Both *Pelagibacterales* and *Rhodobacterales* clustered apart together from all other taxa when using the Comb-DB. Regarding the functional clustering, the 60 kDa chaperonin was found to stand out all other functions apart from NAM-DB in the gel-based approach. Despite the similar trend observed for the most abundant taxa and most represented protein functions for all metaproteomes, Figure 4 clearly shows that the methodology was found to significantly alter the structure/function network.

**FIGURE 5:**
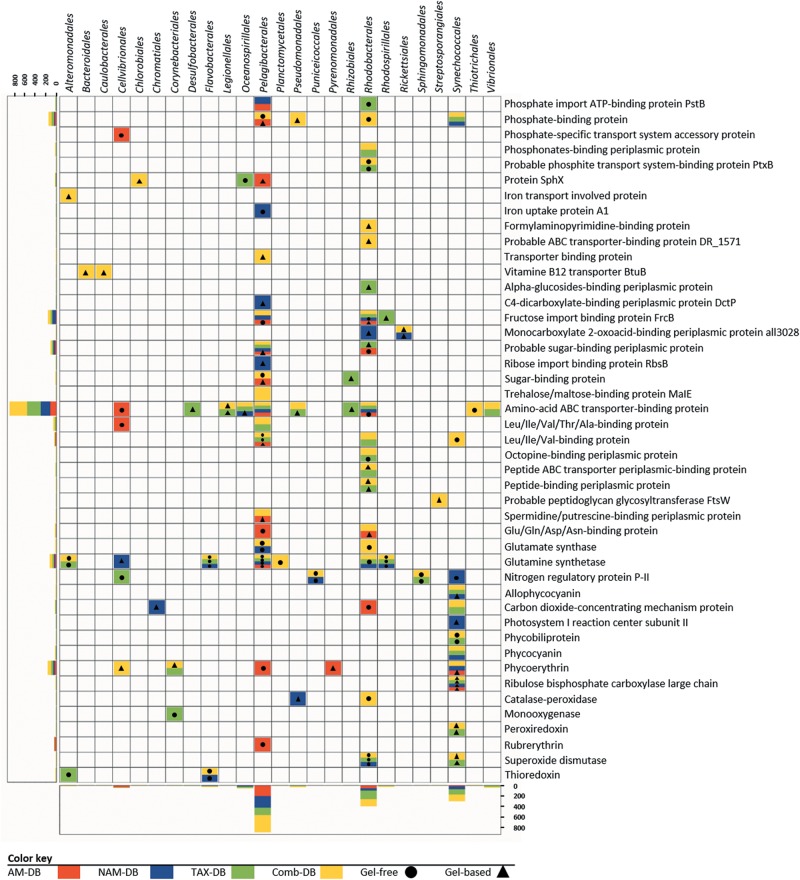
Diversity and taxonomic distribution of proteins involved in nutrient transport, nitrogen assimilation, light harvesting, and oxidative stress response for each methodology. Horizontal and vertical bar charts correspond to the total number of peptides detected for a given function (*y*-axis) or order (*x*-axis) in all metaproteomes. Protein isoforms and/or sub-unit were grouped under the same function. The lack of symbol in colored boxes means that the protein was observed in both gel-free and gel-based approaches.

Interestingly, the detection in all metaproteomes of numerous transporters, mainly for amino-acid/peptide and carbohydrate substrates, across different taxa demonstrated the strategy evolved by bacteria to survive under nutrient-limited environments (Figure 5) (Button and Robertson, 2000; Zimmer et al., 2000; Hoch et al., 2006; Williams and Cavicchioli, 2014). In contrast, key proteins involved in iron, nitrogen, phosphorous, or vitamin metabolisms were characterized in only few metaproteomes. These results emphasized the risk of misinterpretation on the bacterial response to oligotrophic conditions.

The detection of proteins involved in light-harvesting, photosynthesis, and oxidative stress response was found to be particularly dependent of the workflow (Figure 5). A total of 16 of the 26 proteins were characterized in only one metaproteome, showing that a robust experimental design using multiple methodologies will improve the understanding of the microbial light response. Indeed, combining the information found in all metaproteomes helped at depicting the variety of pigments belonging to photoautotrophs or photoheterotrophs (Giovannoni et al., 2005). The characterization of the carbon dioxide-concentrating mechanism protein Ccmk together with the ribulose bisphosphate carboxylase (RuBisCO) informed on how primary producers, such as *Synechococcales* and *Rhodobacterales* overcome inorganic carbon limitation (Woodger et al., 2003; Sowell et al., 2009). Overall, several oxidative stress-related proteins and numerous chaperonin proteins were identified in all metaproteomes, suggesting the adaptability of the microbial community to cope with oxidative stress. As a reminder, surface water samples were collected in summer at the surface of the Mediterranean Sea, where high solar irradiance was encountered. Chaperones are essential for coping with UV-induced protein damage and maintaining proper protein function (Matallana-Surget et al., 2013). Consequently, those metaproteomics results suggest that strategies used by microorganisms to cope with high solar radiation could be similar to the ones extensively described in axenic cultures using microcosms experiments (Matallana-Surget and Wattiez, 2013).

## Conclusion

Metaproteomics enables to progress beyond a mere descriptive analysis of microbial community diversity and structure, providing specific details on which bacteria, and which pathways of those key players, are impacted by possible perturbations. Nevertheless, using this powerful tool without fully apprehending the limitations could lead to significant misinterpretations, especially in the case of comparative metaproteomic studies. This study clearly evidenced the implications of critical decisions in metaproteomic workflow. Our findings lead to the general recommendation of diversifying when possible the protein search database as well as protein fractionation, especially if only one condition/ecosystem was studied. A robust diversified workflow allows crossing information from multiple metaproteomes in order to accurately describe the functioning of microbial communities. In a comparative metaproteomic study however, the best compromise relies on the creation of a Comb-DB. Our findings will undoubtedly serve future studies aiming at reliably capturing how microorganisms operate in their environment.

## Data Availability Statement

Publicly available datasets were analyzed in this study. This data can be found here: PXD014582.

## Author Contributions

SM-S conceived the study, performed the water sampling, and protein extraction. SM-S and RW performed mass spectrometry analysis. AG, JW, and SM-S participated in the design of the mPies program. JW developed the mPies program. AG analyzed all the data and wrote the manuscript. AG and JW prepared the figures. SM-S, RW and PL contributed the resources. All authors edited the manuscript and approved the final draft of the manuscript.

## Conflict of Interest

The authors declare that the research was conducted in the absence of any commercial or financial relationships that could be construed as a potential conflict of interest.
