# Supplementary material for: Deciphering the Functioning of Microbial Communities: Shedding Light on the Critical Steps in Metaproteomics
Source: Front Microbiol. 2019 Oct 24;10:2395. doi: 10.3389/fmicb.2019.02395 (PMC6821674; doi:10.3389/fmicb.2019.02395)
Supplement: Supplementary file 2 [file Image_1.pdf]

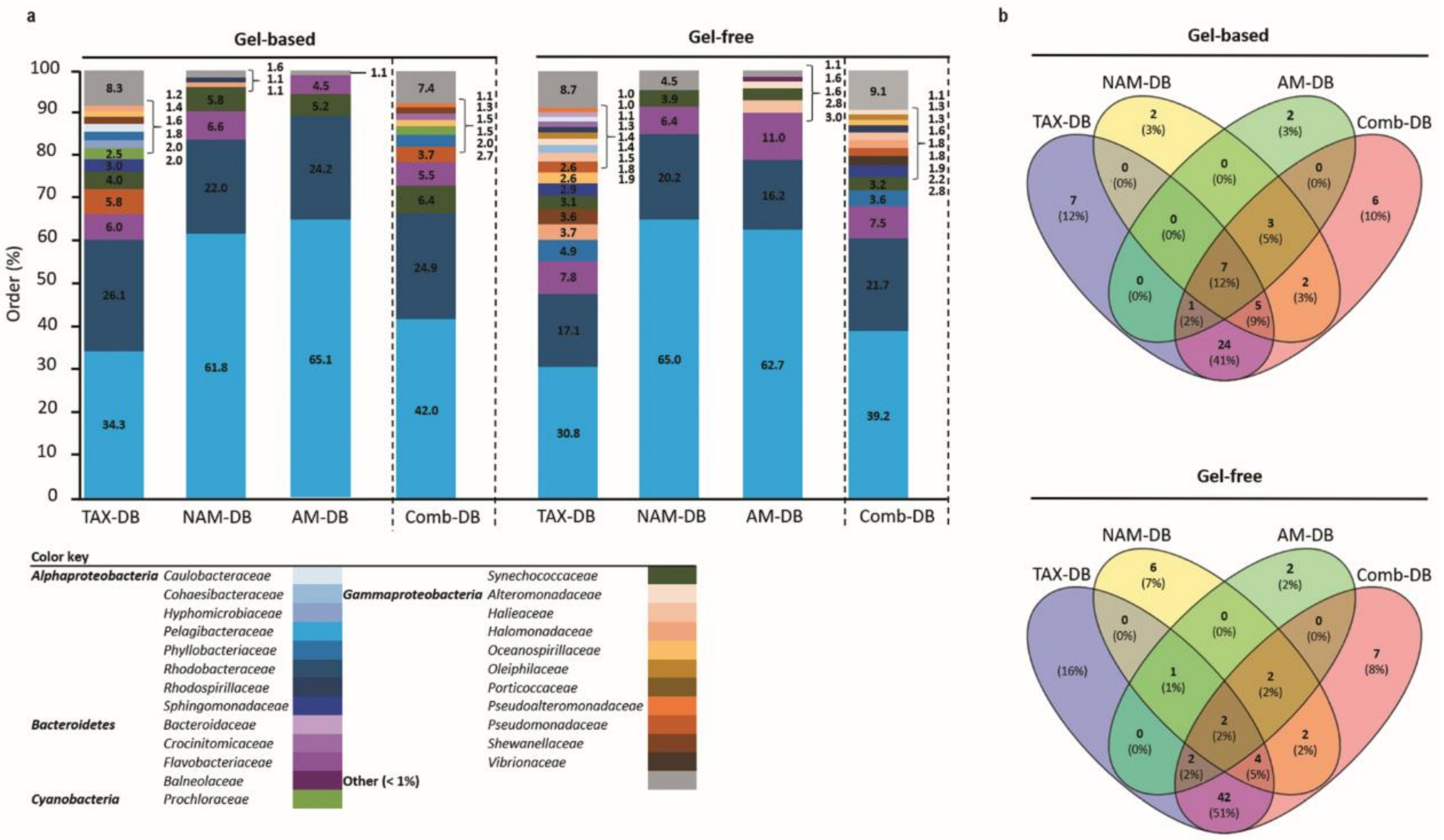

(**a**) Relative taxonomic composition at family level for each methodology. Values represent the proportion of proteins with identical taxonomy on total identified protein using TAX-DB, NAM-DB, AM-DB or Comb-DB in both gel-free and gel-based approaches. The number of peptides detected for each protein was used as quantitative value. Taxa displaying a proportion < 1% were gathered into "Other" category. (**b**) Venn diagrams showing the number of common and unique taxa identified at family level.
