# Supplementary material for: Deciphering the Functioning of Microbial Communities: Shedding Light on the Critical Steps in Metaproteomics
Source: Front Microbiol. 2019 Oct 24;10:2395. doi: 10.3389/fmicb.2019.02395 (PMC6821674; doi:10.3389/fmicb.2019.02395)
Supplement: Supplementary file 4 [file Presentation_1.pdf]

mPies program features

- Project name: mPies: a novel metaproteomics tool for the creation of relevant protein databases and automatized protein annotation (DOI: 10.1101/690131).
- Project home page: https://github.com/johanneswerner/mPies
- Archived version: 10.5281/zenodo.2539710
- Operating system(s): Linux
- Programming language: Python 3
- Other requirements: Conda, other packages installed with mPies: CD-Hit AuxTools (10.1093/bioinformatics/bts565), Diamond (10.1038/nmeth.3176), ETE3 (http://mbe.oxfordjournals.org/content/early/2016/03/21/molbev.msw046), FragGeneScan (10.1093/nar/gkq747), MEGAHIT (10.1093/bioinformatics/btv033), Prodigal (10.1186/1471-2105-11-119), Snakemake (10.1093/bioinformatics/bts480), Spades (10.1089/cmb.2012.0021), Trimmomatic (10.1093/bioinformatics/btu170). At least 150 GB of disk space (depending on the used database), 64 GB memory (if assembly is necessary) and 16 CPUs are recommended for running mPies.
- License: GNU GPL-3.0
- Any restrictions to use by non-academics: none
