# Supplementary material for: Deciphering the Functioning of Microbial Communities: Shedding Light on the Critical Steps in Metaproteomics
Source: Front Microbiol. 2019 Oct 24;10:2395. doi: 10.3389/fmicb.2019.02395 (PMC6821674; doi:10.3389/fmicb.2019.02395)
Supplement: Supplementary file 5 [file Presentation_2.pdf]

Examples of functional consensus annotations returned using mPies based on the 20 best blast hits. The three examples were retrieved using second-round search NAM-DB in gel-free approach.

Example A: automatic mPies annotation

Candidate annotations from the top alignment hits	Frequency	Similarity (%)
Glutamine synthetase	16	80
Glutamine synthetase nodule isozyme	1	5
Glutamine synthetase root isozyme 3	1	5
Glutamine synthetase root isozyme A	1	5
Glutamine synthetase root isozyme B	1	5

Consensus functional annotation

Glutamine synthetase

Example B: manual annotation

Candidate annotations from the top alignment hits	Occurrences	Proportion (%)
60 kDa chaperonin 1	9	45
60 kDa chaperonin 2	3	15
60 kDa chaperonin 3	3	15
60 kDa chaperonin	2	10
60 kDa chaperonin 4	1	5
60 kDa chaperonin 5	1	5
60 kDa chaperonin 7	1	5

Consensus functional annotation

60 kDa chaperonin

Example C: no consensus annotation

Candidate annotations from the top alignment hits	Occurrences	Proportion (%)
3-hexulose-6-phosphate synthase	2	25
5,6,7,8-tetrahydromethanopterin hydro-lyase	2	25
5,6,7,8-tetrahydromethanopterin hydro-lyase	2	25
Bifunctional enzyme Fae/Hps	2	25

Consensus functional annotation

NA
